# Himalayan Sources of Anthocyanins and Its Multifunctional Applications: A Review

**DOI:** 10.3390/foods12112203

**Published:** 2023-05-30

**Authors:** Mustafa Ahmed, Ipsheta Bose, Gulden Goksen, Swarup Roy

**Affiliations:** 1 School of Bioengineering and Food Sciences, Shoolini University, Solan 173229, India; mustafaahmed9560@gmail.com (M.A.); ipsheta18@gmail.com (I.B.); 2 Department of Food Technology, Vocational School of Technical Sciences at Mersin Tarsus Organized Industrial Zone, Tarsus University, 33100 Mersin, Turkey; guldengoksen@tarsus.edu.tr; 3 Department of Food Technology and Nutrition, School of Agriculture, Lovely Professional University, Phagwara 144411, India

**Keywords:** anthocyanin, himalayan plant, natural colorant, food, nutraceuticals, smart packaging

## Abstract

Anthocyanins, the colored water-soluble pigments, have increasingly drawn the attention of researchers for their novel applications. The sources of anthocyanin are highly diverse, and it can be easily extracted. The unique biodiversity of the Himalayan Mountain range is an excellent source of anthocyanin, but it is not completely explored. Numerous attempts have been made to study the phytochemical aspects of different Himalayan plants. The distinct flora of the Himalayas can serve as a potential source of anthocyanins for the food industry. In this context, this review is an overview of the phytochemical studies conducted on Himalayan plants for the estimation of anthocyanins. For that, many articles have been studied to conclude that plants (such as *Berberis asiatica*, *Morus alba*, *Ficus palmata*, *Begonia xanthina*, *Begonia palmata*, *Fragaria nubicola*, etc.) contain significant amounts of anthocyanin. The application of Himalayan anthocyanin in nutraceuticals, food colorants, and intelligent packaging films have also been briefly debated. This review creates a path for further research on Himalayan plants as a potential source of anthocyanins and their sustainable utilization in the food systems.

## 1. Introduction

Nature has lavishly gifted mankind with a plethora of medicinal herbs that have long served as a provider of conventional medicine to treat many human illnesses. Plant natural products are a diverse class of chemical entities with extensive structural and chemical diversity, as well as biochemical specialization and a wide range of biological activity. These have been utilized as medications, additives, insecticides, agrochemicals, fragrance and flavor components, food additives, and pesticides with great care. For thousands of years, plants have served as the basis for many traditional medicines used around the world, and they continue to provide people with novel treatments. They are one of the most abundant sources of chemicals. The complex relationship between plants and people has a long evolutionary history. For centuries people have used plants for food, fiber, medicines, and energy [1]. In the twenty-first century, plants are being used to derive pharmaceuticals, multicomponent herbal products, nutraceuticals, and functional foods [2]. 

The Himalayas, one of the youngest and largest hill systems on this planet, is well known for its floral and faunal diversity [3]. The Himalayan biodiversity hotspot is shelter to a high percentage of flora and animals, along with several environmental assets, yet the people who live here struggle to meet basic requirements such as food and nourishment. Wild plants and their fruits, on the other hand, contribute greatly to the existence of the indigenous communities in the Himalayan region. Plants contain phytochemicals which are secondary metabolites produced for disease protection and contribute to their color, aroma, and flavor [4]. Phytochemicals have multiple uses in pharmaceuticals and agricultural products and as coloring agents and additives in the food industry [5]. One of the most numerous and widespread phytochemicals are flavonoids. These are a category of health-promoting phytochemicals with variable phenolic structures. Based on their chemical structure, flavonoids are further assorted into several subgroups which include flavones, chalcones, flavonols, isoflavones, and anthocyanins [6], of these subgroups, this review is majorly focused on anthocyanins. 

Anthocyanins are a class of water-soluble pigments (colored substances) present in plant sources, particularly fruits and vegetables. They are responsible for giving fruits, vegetables, and tubers their various colors, such as purple, red, and blue. These pigments from the phenol group have been glycosylated. The term ‘Anthocyanins’ originated from ancient Greek language words ‘Anthos’ and ‘Kyanous’. The first implies flowers, while the second implies a deep blue color [7]. Many millennia ago, anthocyanins were consumed by humans through the means of dried leaves, fruits, roots, or seeds and were employed in traditional herbal treatments utilized by North American Indians, Europeans, and Chinese. For decades, anthocyanins have been a part of traditional herbal medicine due to their highly valued medicinal properties. Historically, anthocyanin-rich mixes and extracts were used for treating hypertension, pyrexia, liver diseases, diarrhea, dysentery, and ailments of the urinary system such as renal calculi, urinary tract infections, and rhinitis (common cold). Anthocyanins have wide applications, particularly in the food industry due to their characteristic property of imparting vibrant colors. Food color is an important sensory property that impacts consumer preference. Keeping in mind the safety concerns pertaining to the practice of using synthetic food colorants such as lemon yellow, the use of nature-derived compounds such as anthocyanins and their acylated counterparts as food colorants have become more popular owing to their edible nature and possible health benefits. Several studies on the exploration and utilization of anthocyanins and their related acylated variants as food colorants were conducted. Because the color of anthocyanins and their related forms depends mainly on the surrounding pH conditions, they were investigated as pH-sensitive dyes for their utilization in food packaging. Since food decomposition is generally followed by a change in pH, variations in the tints of sensitive anthocyanin dyes can be used for the determination of the quality and stability of packaged foods [8]. Choi et al. created an intelligent pH-sensitive color indicator by adding anthocyanin pigment extract from purple sweet potato on a matrix of agar-starch and utilized it to detect pH variations in meat products by evaluating the colors at various pHs as extent of deterioration. The color of the indicator, which ranges from red to green, may represent the degeneration of the meat due to pH fluctuations. Anthocyanins have also been added to food products and demonstrating their physiological effect is a field of ongoing research [9]. 

Because of their enhanced stability and fat solubility, anthocyanins have proven to be capable of being used as a food dye, functionalization, and active and smart packaging in food processing. However, there are still challenges and limitations regarding the stability and widespread use of these pigments in food processing industries. Taking the published studies into account, this review aims to compile available information concerning plant sources of anthocyanins distributed across the Indian Himalayan region with a special focus on the multifunctional applications of anthocyanins obtained from these sources. Additionally, some emphasis has been given to their chemical, physical and functional properties, and extraction techniques. The contemporary review critically examines the outcomes of phytochemical studies published on Himalayan plants. There are many works published on anthocyanin and its application but based on the literature survey we concluded there is no review report published on Himalayan anthocyanin and insight into this work is expected to offer more interest in exploring the potential of unexplored anthocyanin from nature and its use in multi-dimension application.

## 2. Chemistry of Anthocyanins

**Figure 1:**
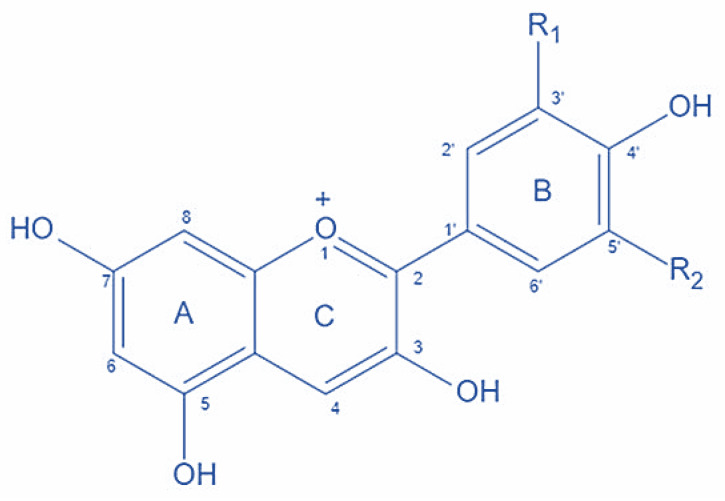
Structure of anthocyanin.

The structure of anthocyanin is given in Figure 1. The C6-C3-C6 unit (xanthine cation) carbon skeleton of anthocyanins is made up of anthocyanidin (aglycone units) coupled to sugar, which is often present at the 3-position on the C-ring, as well as hydroxyl and methoxyl groups [10]. However, the substitution pattern in the B-ring has a significant impact on anthocyanin stability, which can either become better with more methoxyl groups or develop worse with more hydroxyl groups. 

Anthocyanins are hydrophilic natural food colorants with molecular weights of around 400 to 1200 Da (g/mol). In polar solvents such as methanol, ethanol, and water, anthocyanins are soluble. Anthocyanin’s chemical makeup is based on the salts of the flavylium (2-phenyl benzopyrylium) glycoside. Anthocyanin’s structure can be diverse, reliant on the nature, position, and the total aliphatic or aromatic acids involved in the sugars. Commonly, anthocyanins have a sole glucoside unit, nevertheless, there are several types of anthocyanins with two or multiple sugars bound at several positions or involved as oligosaccharide side chains. The stability of anthocyanins can be increased through acylation and glycosylation. Additionally, anthocyanins can be affected by pH, enzymes, light, chemical structure, solvents, oxygen, heat, concentrations, and a few other factors which may influence their stability [11]. Acylated anthocyanins are prospective substitutes for anthocyanins due to their distinctive properties and added benefits over non-acylated counterparts. Acylation brings a structural change in anthocyanins that alters how they function [12,13,14]. Acylated anthocyanins have complicated acylation and glycosylation patterns. They occur naturally in many plants [15]. However, anthocyanins can also be acylated in vitro by chemical and enzymatic methods. These in-vitro acylated anthocyanins are more stable and highly soluble in organic solvents as compared with non-acylated anthocyanins [14]. It has been found that deterioration by heat (thermal degradation) has a damaging result on anthocyanins, which mainly depends on their structure [16]. Thermal degradation of anthocyanins during food processing is a major problem for the industry [17]. Anthocyanins can initially release glycosyl moieties on heating. The resulting anthocyanin aglycone is subsequently broken down into phenolic acids and phloroglucinaldehyde [18]. Heat-induced degradation kinetics depend on the specific chemical makeup of anthocyanins and the properties of the food matrix. For instance, degradation kinetics can be altered by the presence of phenols, ascorbic acid, micellar systems, and soluble solids [19]. 

Due to the instability and ease of degradation, the use of anthocyanins in a wide spectrum of applications is restricted. As a result, it is difficult to use anthocyanins in food products on an industrial scale [20]. Anthocyanin-containing foods can only go into the blood circulation for better absorption and metabolism after passing through the lumen of the intestine. As a result, improving anthocyanin stability is required to optimize metabolism and absorption and consequently upgrade their health merits. Anthocyanins have been reported to be protected against unfavorable environmental conditions by encapsulated delivery systems [21]. One of the most significant challenges in manipulating anthocyanins is that they are extremely susceptible to deterioration, especially when separated. Because several physicochemical factors have been shown to interact with anthocyanin stability, basic anthocyanin chemistry is needed to comprehend the limitations of a certain extraction procedure. 

## 3. Extraction of Anthocyanins

Various traditional and developing procedures have been used to extract anthocyanins from plant sources. Each extraction method is unique and has a profound result on the stability, purity, yield, and concentration of the extracted anthocyanins [22]. Natural anthocyanins are particularly unstable and prone to degradation, resulting in loss of bioactivity and color. Factors affecting the rate of decomposition include heat, light, oxygen, pH, enzymes, co-pigments, and water activity [23]. In the literature on anthocyanins, two main extraction processes were discovered: methods that optimize the extraction to define and detect anthocyanins, and methods that are expandable for application in the food sector [24]. Several factors, particularly the solvents, must be considered in the anthocyanin analysis. Due to their polarity, anthocyanin molecules can be extracted using a variety of polar solvents such as methanol, alcohol, and acetone water. Since anthocyanins are highly reactive compounds, choosing an appropriate solvent for extraction is critical. Traditional techniques for extracting anthocyanin include soaking, stirring, ultrasound-assisted extraction, and enzyme-driven extraction. The growing interest in anthocyanin’s antioxidant activity drives a greater desire for more efficient extraction methods, such as lower solvent usage, lower environmental repercussions, higher extraction yield, and shorter extraction timeframes [25].

## 4. Himalayan Sources of Anthocyanins

**Figure 2:**
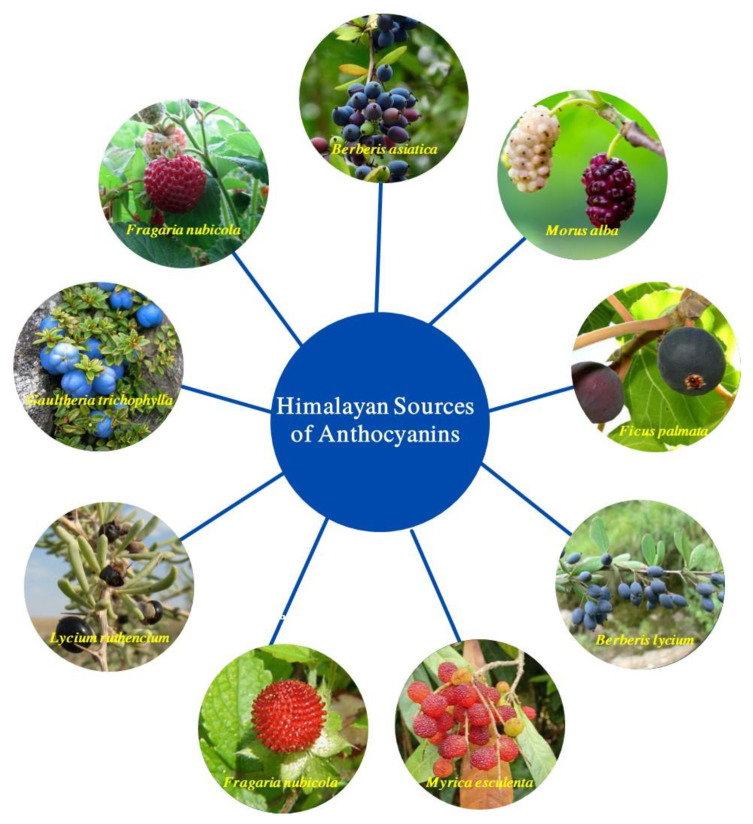
Various sources of plant-based Himalayan anthocyanin.

Himalayan plants are excellent sources of anthocyanin. Fruits, flowers, leaves, and roots of plants are natural sources of anthocyanins. Cherries, berries, and cereals are among the most popular anthocyanin found in natural goods. Numerous anthocyanins and their compounds extracted from natural sources have been mentioned in the scientific literature. A brief discussion of anthocyanins from Himalayan sources is provided below. Figure 2 shows some of the indigenous Himalayan sources of anthocyanin. On the other hand, the classification of various Himalayan anthocyanins is presented in Table 1.

### 4.1. Berberis asiatica

With more than 500 species found worldwide, the genus Berberis is widely recognized for its therapeutic potential [34]. *Berberis asiatica* is the most widely distributed species in the Western Himalayas and Northeast India. It is found in Jammu and Kashmir, Himachal Pradesh, Uttarakhand, Arunachal Pradesh, and Assam. 

*Morphological aspects*: It is an evergreen shrub with a height of 1.2–1.8 m and the stem is up to 10 cm in diameter. It has a rough, furrowed, and corky bark. Twigs are yellowish with a smooth surface. Leaves are dark green, oblong, elliptic, and usually have large distant spinous teeth. The size of the leaves is 2.5–6.3 by 1.3–3.8 cm. Prominent primary and secondary pale reticulate venation above and glaucous beneath can be observed. Petiole is usually absent but sometimes visible up to 10 mm. Simple racemose type of inflorescence which is 3 cm long. Pedicels are 4–19 mm long, slender, often glaucous. This plant yields fruits that are 7–10 mm long, ovoid, and dark black in color [35]. 

*Functional and therapeutic roles*: The root is widely utilized in many indigenous therapeutic practices to treat a wide range of health problems such as rheumatism, diabetes, jaundice, gastric disorders, skin conditions, malarial fever, and as a tonic, among others. The presence of phenols in this plant is thought to be responsible for its wide range of health-promoting properties. These phenols provide antioxidant action through a variety of mechanisms, including the removal of free radicals, metal chelation, hydrogen donors, and gene expression alterations [36]. The fruits are eaten raw and taste juicy with an acidic flavor. Traditionally, these fruits are provided to children as mild laxatives and the juice may be helpful in dental ailments. The species is employed in pharmacological, nutraceutical, and cosmetic products [37].

*Phytochemical aspects*: The *Berberis asiatica* plant is a reservoir of various bioactive chemical compounds such as phenolics, alkaloids, flavonoids, anthocyanins, tannins, vitamins, and minerals [38]. As per physicochemical investigations, it was revealed that berberine, an alkaloid found in the plant was 2.4%, tannins 1.73%, total ash 2.650%, starch 16.444%, acid insoluble ash 0.266%, and alcohol soluble extractive 11.833%. [35]. Berberine is the plant’s main alkaloid, it is a quaternary ammonium salt that comes under the protoberberine class of isoquinoline alkaloids. It is known for its biological activity against many chronic diseases [39]. 

*Anthocyanins*: The anthocyanin content expressed as mg of cyanidin-3-glucoside equivalents (CGE) per g of fresh weight (mg CGE/g fw) in these fruits were found to be 24.59 mg/100 g fw [40]. To date, there are very limited studies available on the anthocyanin composition of the *Berberis asiatica* plant. 

### 4.2. Morus alba 

The Genus, *Morus* (Mulberry) is a genus of flowering plants with approximately 150 species of which *Morus alba* L. is the most important [41]. It is a plant with significant economic value that is extensively cultivated in Centra, East, and South Asia for sericulture. *M. alba* species are thought to have evolved on the low Himalayan slopes that border China and India [42]. With around 626,000 hectares of land under Mulberry cultivation, China comes first in terms of Mulberry production, followed by India at 280,000 hectares of land under cultivation [43]. 

*Morphological aspects*: It is a deciduous tree of medium-sized found in China and hilly areas of the Himalayas at an elevation of up to 3300 m. The tree grows up to a height of 20–30 m and a girth of 1.8 m. It can be reduced to a low-growing bush to make it easier to gather leaves and fruits. Dark grey-brown lenticels run horizontally down the bark. The petioles are long and thin, the leaves are alternating, glossy green collects at the apex, and the margins are notched or toothed at the end (serrated). The leaves’ lengths range from 5.0 to 7.5 cm, and they have a wide range of shapes. It has been observed that the tress are dioecious in temperate and sub-tropical climatic zones but in some cases, they change from one sex to another. Male and female catkins are made up of discrete, pendulous, greenish blooms. The characteristic feature of the mulberry fruit is that it has multiple drupes formed by each flower to make a sorosis. The weight of the fruit is approximately 3.49 g with a moisture content of nearly 71.5%. The color of the fruit varies with its maturity. In the early stages, the color is green which turns orange to red in the intermediate stage. When it is fully matured, the color is purplish black [44,45,46]. 

*Functional and therapeutic roles*: Traditionally, it is used in kidney disorders and purification, curing weakness, anemia, fatigue, and early graying of hair. Due to its calming properties, the plant is beneficial in monitoring illness according to Chinese traditional medicine. It functions by reducing the symptoms of sore throat, cough, and fever, protecting the liver, enhancing vision, helping in urination, and stabilizing blood pressure [47]. The *Morus alba* plant has been also known to show excellent therapeutic activity against diabetes mellitus due to the presence of a special alkaloid 1-deoxynojirimycin (DNJ) [48].

*Phytochemical aspects*: It has been revealed that *Morus alba* majorly contains alkaloids (Calystegins B2, C1, and 1-Deoxynojirimycin), terpenoids (betulinic acid, ursolic acid, and uvaol), flavonoids (astragalin, atalantoflavone, anthocyanins and chalcones such as Morachalcones B, C), phenolic acids, stilbenoids, and coumarins [44]. These bioactive components make it highly beneficial for multifunctional applications.

*Anthocyanins:* The content of anthocyanin in the fruit was found to be 21.97 mg CGE/100 g fw [40]. 

### 4.3. Ficus palmata

The common wild fig (*Ficus palmata*) grows on hot, dry slopes of Northwest hills, on clay-loam soils in the states of Uttarakhand, Punjab, and Kashmir in India, Nepal, the Arabian Peninsula, Ethiopia, Somalia, Sudan, Pakistan, Afghanistan, Iran, and Southern parts of Egypt [49]. 

*Morphological aspects*: It is a deciduous tree with an average height of 6 to 10 m. Leaves are broad, ovate, alternate, and membranous with a size of around 12.93 cm long and 14.16 cm broad. It has small flowers which are unisexual, monoecious, and greenish-white in color. It yields deep violet to black colored fruits having a diameter of approximately 2.5 cm and measuring 6 g in weight. Both fruit and seeds are edible. Due to the presence of white latex below the fruit’s outer covering, the fruit may have a little acidic or bitter taste and smell (astringency) [50].

*Functional and therapeutic roles*: In the Uttarakhand region of the Indian Himalayas, fruits of *Ficus palmata* are well-known for their traditional use in treating ailments such as stomach ulcers, digestive problems, bronchitis, eczemas, hemorrhoids, and as a diuretic. In traditional medicine, *Ficus palmata* is commonly used for its digestive, hypoglycemic, insulinase, anti-tumor, anti-ulcer, antidiabetic, lipid-lowering, anti-carcinogenic, antifungal, and inflammation-reducing properties [51]. It can also be used as a dietary component in the treatment of constipation and ailments related to the gallbladder and respiratory system. The plant’s sap is often administered as the medication for warts [50]. 

*Phytochemical aspects*: Numerous phytoconstituents, including alkaloids, steroids, lipids and fixed oils, flavonoids, tannins, proteins, and sugars, are found in the bark, root, and fruits. It is a promising source of flavonoids, polyphenolic compounds with powerful antioxidant qualities that aid in the prevention and treatment of a variety of oxidative stress-related disorders, including neurological and hepatic conditions [52]. 

*Anthocyanins*: The anthocyanin content in the fruits was estimated to be 19.27 mg CGE/100 g fw [40]. 

### 4.4. Berberis lycium

*Berberis lycium* is a native plant to the Himalayan mountains and is extensively dispersed in temperate and semi-temperate climatic zones of India, Nepal, Bangladesh, and Pakistan. 

*Morphological aspects*: It has strong woody branches covered by thin, brittle bark. The plant is a small-sized, stiff deciduous shrub that can reach heights of 1.0 to 2.5 m. Leaves are 2.5 to 7.5 by 8.18 mm in size and pale green in color. The plant’s stem has a few noticeable spinous teeth that are alternately placed. The inflorescence is composed of corymbose racemes, which have 11–16 flowers per cluster. Insects pollinate flowers, which are often hermaphrodite, borne in axillary clusters, and pale yellow in color. The plant’s blooming and fruiting season lasts from March through July. The first two weeks of March mark the start of the flowering season, which lasts until April. In the second week of May, the fruit starts to ripen, and it does not fully mature until June. Fruits are incredibly nutrient-rich in the form of 8 mm long globose ovoid berries with bluish-purple color when they are fully mature [53,54].

*Functional and therapeutic role*: Since the beginning of time, ancient societies of the Himalayan region of Jammu and Kashmir, and Himachal Pradesh have utilized these berries as a food source [55]. The herb is employed in traditional medical practices to cure several ailments and disorders. People use the various parts of the plant, such as the leaves, stem, roots, fruits, and flowers, as food and medicines. The plant is well known for preventing diseases of the skin, eyes, and abdomen. Recent research work has revealed that it has good antimicrobial, antidiabetic properties [56]. 

*Phytochemical aspects*: Berberine, palmatine, and jatrorrhizine are the major alkaloid found in this species with berberine being the most prominent one. It was discovered that the root extract of *B. lycium* contained 80% dry weight of berberine and very small levels of other alkaloids [57,58]. There are several important flavonoids and phenolic compounds present in this plant, such as chlorogenic acid, hydroxybenzoic acid, quercetin, rutin, and mandelic acid. Ascorbic acid, β-carotene, and anthocyanins are also found in the fruit in significant amounts [59]. 

*Anthocyanins*: The *B. lycium* fruit’s anthocyanin content was reported to be 20.58 mg CGE/100 g fw [60]. Characterization of *Berberis lycium* anthocyanins by LC-MS and UV spectral analysis revealed that there are a total of twelve anthocyanins in the purified extract, but among all the anthocyanins, delphinidin-3-glucoside (35.3%) and cyanidin-3-glucoside (47.2%) were the most prominent. The glycosides of cyanidin, pelargonidin, malvidin, and peonidin were the other ten anthocyanins that were also characterized. Overall, it was discovered that the species has a potential future for use in food systems [61]. 

### 4.5. Myrica esculenta

In India, the genus Myrica is represented by *Myrica esculenta* species [62]. It is an evergreen dioecious small tree of Indian origin and is widely disseminated along the mid-Himachal Pradesh foothills track, which extends from Ravi eastward to Assam and includes the states of Sikkim, Manipur, Arunachal Pradesh, Uttaranchal, and the Khasi, Naga, Jaintia, and Lushai Hills of Meghalaya, all of which are located between 900 and 2100 m above sea level. It is also found in Singapore, China, Pakistan, Japan, Nepal, and the Malayan Islands [63]. 

*Morphological aspects*: It is a medium to large woody tree with a trunk diameter of 92.5 cm and a height between 12 and 15 m. Most leaves are clustered at the ends of branches and are lanceolate with an entire or serrated edge, pale green on the underside, and dark green on the top side. While the inflorescence of staminate flowers is a compound raceme, the inflorescence of pistillate flowers is small, sessile, solitary, and bracteate; the sepals and petals are either absent or not visible; the catkin’s axillary length is 4.2 cm; it bears about 25 flowers in a thread-like fashion. The flowering season begins in February and lasts through the second week of April, however, the first week of March marked the peak flowering period. Fruits are drupe, red to dark brown in color, oval, and about 2–7 mm in diameter. Fruits taste sweet and sour [29]. 

*Functional and therapeutic roles*: Local population use this traditional ayurveda plant in a variety of ways due to the diverse therapeutic benefits of the plant parts. Natural antioxidants included in *M. esculenta* fruit are a significant source of protection against oxidative stress and certain degenerative illnesses [64]. Research has demonstrated that flavonoids and phenolic acids found in the plant are responsible for its antioxidant potential. Analgesic, antiasthmatic, anticancer, antidepressant, antidiabetic, anti-inflammatory, chemopreventive, hepatoprotective, and wound healing effects are some of the pharmacological activities that have been reported recently [65]. Moreover, they are non-toxic and have no harmful materials which makes them useful for versatile applications.

*Phytochemical aspects*: *Myrica esculenta* plant has been discovered as a rich source of flavonoids, flavonols, and phenolic chemicals. Other bioactive components of the plant include alkaloids, glycosides, diarylheptanoids, ionones, steroids, saponins, triterpenoids, and volatile compounds. [29,63]. Myricetin and quercetin are the major flavonoids whereas flavonoid glycosides such as Myricitrin (myricetin 3-O-rhamnoside) were also detected. Arjunolic Acid was the major saponin. Corchoionoside C; (6S,9R)-roseoside was the ionone [66,67]. 

*Anthocyanins*: The fruit had an anthocyanin content of 7.17 mg CGE/100 g [60]. The major anthocyanins detected were malvidin 3-(6″-acetylglucoside), cyanidin 3-O-(6″-acetylglucoside), delphinidin-3-O-arabinoside, and cyanidin-3.5-di-O-beta-D-glucoside [68]. 

### 4.6. Duchesnea indica

*Duchesnea indica*, generally known as a mock strawberry/Indian strawberry, is a straggling herb belonging to the family Rosaceae [69]. It is a perennial plant having widespread distribution in Asia, Europe, and America. 

*Morphological aspects*: Stoloniferous species *Duchesnea indica* (Andrews) Focke have bostryx-like cymose inflorescences with incredibly long petioles, yellow petals, terminal styles, anthers with two thecae, and achenes. It can be identified by its perennial habit, long, creeping flower stem, roots in nodes, basal leaves with three to seven lobes, and style that is shorter than or equal to the carpel [70]. Ripened fruits are red, shining, spongy, and 1–2.5 cm in diameter. Achenes are small, ovoid, glabrous, and pitted [71]. Its fruits are also used as a cooling agent, tonic, and in eye infections. This plant’s ash has long been used to cure burns and skin problems. Additionally, leaf water extract has been utilized as an anthelmintic [72]. 

*Functional and therapeutic role*: For thousands of years, it is employed as a traditional herbal medicine in Asia, mostly to cure leprosy, congenital fever, and tissue inflammation. These days, it is clinically employed for cancer treatment or as a key component of formulae for Chinese herbal medicines used to treat cancers, particularly gynecological cancers. A recent report has shown that extracts of the *Duchesnea indica* plant lowered the growth of SKOV-3 ovarian cancer cells by inducing apoptosis via the mitochondrial pathway and stopping cell cycle progression in the S phase [73]. 

*Phytochemical aspects*: This plant has been found to contain a number of phenolic chemicals, such as flavonoids, ellagic acids, and phenolic acids. A total of 27 phenolic compounds have been reported in the species, and they fall into four categories: flavonols, ellagitannins, ellagic acid and its derivatives, hydroxybenzoic acid, and hydroxycinnamic acid. In addition to that, brevifolin carboxylate, caffeic acid, brevifolin, methyl brevifolin carboxylate, and kaempferol O-robinobioside were also reported [74]. 

*Anthocyanins*: The anthocyanin content in its fruit was found to be 7.06 mg CGE/100 g [60]. Another study found the total amount of anthocyanin on a cyanidin-3-rutinoside basis to be 205 mg/g of fresh stoned fruit, with cyanidin 3-O-rutinoside accounting for major composition [75]. The detailed anthocyanin composition has been described in Table 2. 

### 4.7. Lycium ruthencium

Genus Lycium has long been recognized as a source of nutrients and medications throughout Southeast Asia, specifically in China. This genus contains 97 species and 6 variations of perennial flowering plants belonging to the family Solanaceae, which are primarily found in South America, South Africa, China, and a few species in temperate Asia and Europe. *Lycium ruthencium*, locally known as “Khizer”, is a plant that is extensively distributed in the Trans-Himalayan Ladakh region at a height of 3063–3196 m above mean sea level. It grows primarily on the sides of highways in the Nubra valley’s Hunder and Udmaru sections [76].

*Morphological aspects*: It is a thorny, perennial shrub with a long lifespan that is a member of the Solanaceae family. It has small sessile leaves, zigzag-shaped stems, internodes with little thorns, and deep roots. It can reach a height of 2 m. It has pale purple funnel-shaped flowers which are hermaphrodite in nature. The shoots are short with a length of approximately 5–10 mm bearing one or two flowers. The fruits are 6–9 mm long, color ranges from black to purple berries commonly called goji berries. Seeds germinate in the late spring or early summer, while the bushes bloom in the months of June and July and bear fruit in the months of August and September [77]. 

*Functional and therapeutic roles*: These berries are utilized in various herbal remedies as a medication or a coloring component in India [78]. It has been reported that historically, the fruits have been used as treatments for a variety of illnesses, including menstruation-related problems, hypertension, urethral stones, tinea, furuncles, and blindness in camels in mountain communities, particularly in the Chinese and Tibetan medical systems [79,80,81]. While using leaves as a diuretic to clear urine blockage [82].

*Phytochemical aspects*: The primary chemical components that have been identified are alkaloids, phenolic acids, anthocyanins, pro-anthocyanidins, fatty acids, coumarins, polysaccharides, essential oils, carotenoids, and cinnamate derivates. The major bioactive chemical components of *L. ruthenicum* Murr are anthocyanins, which belong to the flavonoids class of phytochemicals and are believed to be mostly responsible for the alleged therapeutic benefits [83]. 

*Anthocyanins*: The anthocyanin content in the fruits was estimated to be 9.28 ± 1.19 to 82.58 ± 0.95 mg CGE per g of dry weight (C3GE/g DW) [77].

### 4.8. Gaultheria trichophylla

*Gaultheria trichophylla* (family-Ericaceae), is a highly valuable wild Himalayan plant. It is a decumbent, aromatic shrub that forms a mat at higher elevations, between 3200 and 5300 m above sea level. Its distribution is restricted to the Trans-Himalayan regions of Pakistan, China, India, and Nepal [84]. The Himalayas are home to this species; hence it is often called ‘Himalayan snowberry’. It produces blueberries that the local community uses as a source of refreshing food. Dried branches are used by people in the Trans Himalaya region of Uttarakhand to make incense fire during religious ceremonies [85]. 

*Morphological aspects*: It is a small herb with a wiry, slender, and dark brown stem of length 4–7 cm. Leaves are oval, with log setae with a dark green upper surface and whitish green lower surface. Leaves are 4–8 mm long with a diameter of 2.3–4 mm. The plant yields oval blue fruits with a length of about 1.4 cm and a diameter of 1 cm [32]. 

*Functional and therapeutic roles*: *Gaultheria trichophylla* fruits have a long history of usage in traditional medical practices, particularly for the treatment of pain and inflammation [32,86].

*Phytochemical aspects*: *Gaultheria trichophylla*’s wild edible fruits have been shown to be extremely nutrient-dense and abundant in polyphenols and antioxidants. The results of the phytochemical analysis show that fruits from the Milam bugyal region have higher levels of total phenolic compounds at 3.71 mg gallic acid equivalents per g of fresh weight (GAE/g FW), flavonoids 1.75 mg quercetin equivalent per g of fresh weight (QE/g FW), tannins 2.62 mg tannic acid equivalent per g fresh weight (TAE/g FW), and flavonols 1.03 mg catechin equivalents per g fresh weight (CE/g FW) [87]. For this plant, so far, no potential studies of its anthocyanin profiling and composition were reported. So, further research needs to be conducted in this area.

### 4.9. Species of Genus Begonia

With 1870 species, the pantropical genus Begonia is the sixth-largest genus of flowering plants. [88]. Begonia is also a significant source of phytochemicals due to its diverse range of taxa and a high degree of morphological variety. Begonia plants have a high content of phenolics such as total phenolic and flavonoids. Begonia is also one of the largest genera of vascular plants, encompassing approximately 1800 species. Additionally, numerous cultivars are grown specifically for their ornamental value as flowers. The northeastern region of India, along with Myanmar, is a significant area for the genus Begonia. Numerous species have been recently described from this region, and many more are currently being studied and evaluated (Taram et al., 2023). Earlier authors have reported the presence of flavonoids in Begonia species. Five flavonols and two flavones were isolated from the leaves of *B. erythrophylla*. These phytochemicals were identified as 3-methyl ethers of kaempferol, 3-methyl ethers of quercetin, quercetin, and its 3-O-rutinoside and 3-O-rhamnoside, and 7-O-glycoside of luteolin. [33]. Additionally, numerous cultivars are grown specifically for their ornamental value as flowers. The red-colored leaves of *B. xanthina*, *B. palmata*, and *B. megaptera* were found to contain a high amount of anthocyanin, which can be used to create biobased color. The amount of anthocyanin in *B. xanthina*, *B. palmata*, and *B. megaptera* was found to be 88.08 mg, 68.26 mg, and 20.08 mg CGE/g fw [89,90]. It is important to conduct comprehensive phytochemical surveys of more wild Begonia species to further explore their anthocyanin content and composition.

### 4.10. Fragaria nubicola

*Fragaria nubicola*, sometimes known as Himalayan strawberry, is a perennial herb that is typically found in shady areas near the edges of forests between the altitudes of 2100 and 4000 m above sea level [31]. Native to the Himalayas, this species is found in Afghanistan, India, Nepal, Tibet, Myanmar, Pakistan, South-Western China, and Bhutan. 

*Morphological aspects*: This plant is a small herb, stoloniferous, with a height of 2–4 cm. Leaves are trifoliate, lateral leaflets are in the form of distinctly petiolate, elliptic, or obovate. Petioles and stems are resisted from spreading. One to numerous flowers can be seen in the inflorescence. The flowering season is from May to August. Flowers are large, sometimes more than 2.5 cm in diameter [91]. Its edible fruits have an anthocyanin-tinged monopodial stolon and are broadly ovoid or compressed ovoid in shape, measuring 5.5–16.5 mm long by 7.0–17.5 mm in diameter [92].

*Therapeutic roles*: Fresh fruits of the plant in combination with dried leaves of *Potentilla peduncularis* and dried roots of *Geumelatum elatum* are made into a fine paste and usually taken orally to treat fever and rhinitis [93]. Tibetan doctors utilize it to treat neuropsychiatric conditions and nerve inflammation [94]. The unripe fruit is chewed to treat acne, while plant juice is applied to ease heavy menstrual bleeding [95].

*Phytochemical aspects*: The plant is abundant in phenolic compounds and ellagic acid, both of which are recognized as powerful antioxidants [96]. Phenolics (1.18–3.08), proanthocyanidins (0.53–1.01), flavonoids (0.99–2.63), flavonols (0.95–1.80), and tannins (0.73–1.42) mg per g gallic acid equivalent (GAE) are the various phytochemicals identified in this plant [97]. 

*Anthocyanins*: The total monomeric anthocyanins in the berries were found to be 1.46 mg CGE/g fw [31].

**Table 2:** Anthocyanin composition of some Himalayan plant sources.

Species	Anthocyanin Composition	Concentration	References
*Berberis lycium*	1. Delphinidin-3-glucoside	35.32%	[61]
	2. Cyanidin-3-lathyroside	0.08%	
	3. Cyanidin-3-glucoside	47.24%	
	4. Cyanidin-3-rutinoside	0.53%	
	5. Cyanidin-3-galactoside	1.62%	
	6. Pelargonidin-3-pentoxilhexoside	2.26%	
	7. Malvidin-3,5-dihexoside	4.21%	
	8. Pelargonidin-hexoside	0.58%	
	9. Pelargonidin-3,5-diglucoside	1.05%	
	10. Cyanidin-3,5-dihexoside	6.12%	
	11. Peonidin-3-rutinoside	0.77%	
	12. Pelargonidin-3-rutinoside	0.22%	
*Morus alba*	Cyanidin-3-glucoside	1. 301.75 mg/g MAE	[98]
	2. Cyanidin-3-rutinoside	2. 108.79 mg/g MAE	
	3. Pelargonidin-3-glucoside	3. NA	
	4. Pelargonidin-3-rutinoside	4. NA	
	5. Cyanidin 3-O-(6″-O-α-rhamnopyranosyl-β-D-glucopyranoside)	5. 270 mg/g CMA	
	6. Cyanidin 3-O-(6″-O-a-rhamnopyranosyl-β-D-galactopyranoside)	6. 57 mg/g CMA	
	7. Cyanidin 3-O-β-D-galactopyranoside	7. 233 mg/g CMA	
	8. Cyanidin 7-O-β-D-glucopyranoside	8. 33 mg/g CMA	
	9. Petunidin 3-O-β-glucopyranoside	9. 33 mg/g CMA	
*Myrica esculen.*	Malvidin3-(6″-acetylglucoside)	0.205 ± 0.4 mg CGE/100 g FW’	[68]
	2. Cyanidin3-O-(6″- acetylglucoside)	0.342 ± 0.4 mg CGE/100 g FW’	
	3. Delphinidin-3-O-arabinoside	0.421 ± 0.4 mg CGE/100 g FW’	
	4. Cyanidin-3.5-di-O-beta-D-glucoside	0.421 ± 0.4 mg CGE/100 g FW’	
*Duchesnea indica*	Cyanidin 3-O-rutinoside	125 mg/g	[75]
	2. Peonidin 3-O-rutinoside	70 mg/g	
	3. Petunidin 3-O-rutinoside	10 mg/g	
*Lycium ruthencium*	Petunidin-3-Orutinoside (trans-p-coumaroyl)-5-O-glucoside	10 mg CGE/g DW	[77]
	2. Petunidin	5.71 mg CGE/g DW	
	3. Malvidin	0.47 mg CGE/g DW	
	4. Delphinidin	0.29 mg CGE/g DW	

MAE = mulberry anthocyanin extract. NA = not available. CMA = crude mulberry anthocyanin. CEE = crude ethanol extract. FW = frozen weight. FW’ = fruit weight. DW = dry weight. CGE = cyanidin-3-glycoside equivalent.

## 5. Physical and Functional Properties

Anthocyanin possesses various important physical and functional properties, and this section briefly discusses it. Reactive oxygen species (ROS), Free radicals, and/or reactive nitrogen species (RNS) are essential for the human body to function normally. Redox homeostasis is responsible for maintaining a balance of these radicals in our body. In certain circumstances, the body may experience oxidative stress brought on by an imbalance of these radicals. This stress results in the progression of chronic degenerative diseases such as cancer, aging, and coronary heart disease [99]. Anthocyanins have been defined as substances that scavenge free radicals and modulate oxidative stress to curb or inhibit oxidation. Anthocyanins function as H-atom donors or as single electron transfer agents. The antioxidant activity of these compounds can be determined using several analytical methods based on both these working mechanisms. The antioxidant nature of anthocyanins is subject to the overall concentration, structure, and environmental conditions [100].

Due to their high reactivity to pH changes, anthocyanins display distinct chemical structures and colors in solutions with varying pH values. Furthermore, as the pH value rises, the absorption peak of anthocyanin solutions in the UV-VIS spectrum exhibits a noticeable bathochromic shift [101]. Anthocyanin-rich solutions turn from red to pink, purple/blue, and yellow as the pH rises. This color change is the result of anthocyanin’s structural transformation from red flavylium cation to purple/blue quinoidal base, colorless carbinol pseudo base, and yellow chalcone [102]. 

Apart from these physical properties, anthocyanins show numerous functional properties in plants, food, and human nutrition. Anthocyanins provide different hues to plants for attracting animals to perform pollination and seed dispersal [103]. Anthocyanins have been known to protect plants from reddening caused by UV-B light. However, hydroxycinnamoylated structures are necessary for absorption to take place in the 280–320 nm region, and many simply glycosylated anthocyanins would not perform this function. More recent research has shown that anthocyanins shield the organelles that contain chlorophyll and thereby avert photoinhibition by protecting chloroplasts against high-intensity lights [104]. Three primary roles for anthocyanins in plants have been proposed: (i) absorbing harmful radiations, (ii) working as modes of transportation for monosaccharides and (iii) as osmotic regulators during drought and low-temperature periods [105,106,107]. Moreover, anthocyanins are regarded as essential phytochemicals for human nutrition [108]. However, the actual health benefits of anthocyanins are determined by their final bioavailability and bio accessibility in the human body. In some clinical studies, individuals were given anthocyanin-rich diets or extracts for consumption, and it was investigated that less than 1% of ingested anthocyanin was available in a standard blood test of those individuals. This study denotes the low bioavailability of anthocyanins [109]. This is attributed to several factors such as (i) degradation in the oral cavity due to interaction with human enzymes, microbiota, and spontaneous binding to salivary proteins, (ii) change in form and stability due to varying pH, water content, and gas composition along the gastrointestinal tract, and (iii) modification by mammalian enzymes during circulation [110,111,112]. Researchers have developed novel techniques such as nano-encapsulation and exosome, polysaccharide-based, lipid-based, and protein-based complexes, for improved bioavailability and controlled release of anthocyanins [113].

## 6. Applications of Anthocyanins

**Figure 3:**
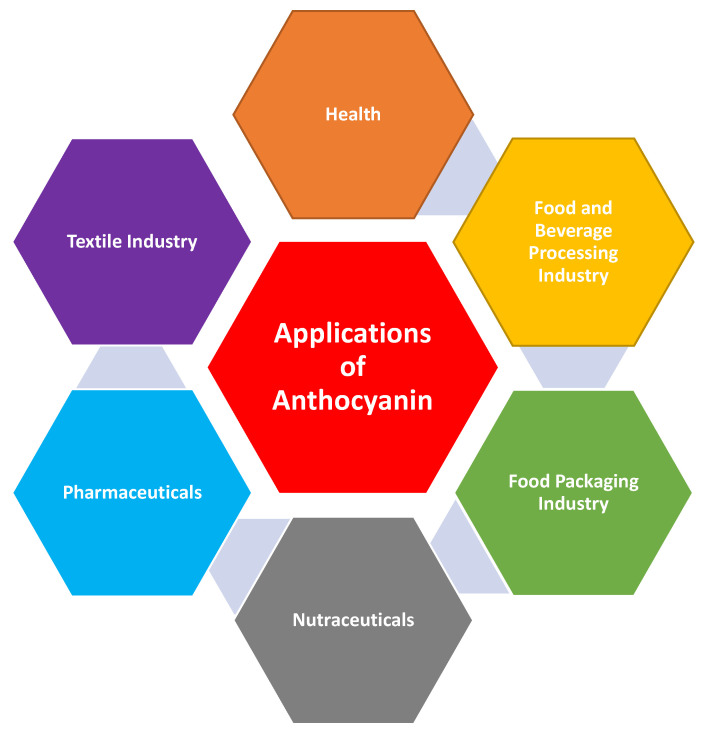
Various applications of anthocyanins.

Anthocyanins have wide applications in the industry, especially as food colorants, as they can produce distinct colors (Figure 3). They also possess exceptional health-promoting properties that have been integrated into human diets for many years. In addition to serving as a source of nutrients, anthocyanins are also found in traditional herbal remedies for conditions such as high blood pressure, pyrexia, liver abnormalities, diarrhea, dysentery, urinary issues, and the common cold [8].

### 6.1. Food and Beverage Processing Industries

The demand for foods and extracts high in anthocyanins have gained attention owing to the health benefits they offer. These extracts can now be used in a wider range of food applications owing to their improved stability and potential health effects [114]. Anthocyanins are extensively being used as natural colorants since synthetic colors used in the food industry were found to have a detrimental impact on human health. Consumers and food manufacturers are searching for natural food colorants over synthetic dyes [115]. According to a 2007 trial conducted on children aged 3 and between 8–9 years, mixtures of artificial colorants such as tartrazine (E102), sunset yellow (E110), carmoisine (E122), ponceau 4R (E124), allura red AC (E129), and quinoline yellow (E104), when combined with sodium benzoate (E211) statistically caused considerable rise of hyperactivity in children [116]. Because synthetic dyes are more stable with regard to light, oxygen, temperature, and pH, the replacement of synthetic dyes with natural food colorants presents certain obstacles [117]. Natural dyes do not particularly have stability and can be characterized by their own physiological activities [118]. Anthocyanins with acylated substituents have been discovered to be more stable during the production and storage of food goods, according to published research. Researchers around the world are evaluating promising sources of acylated anthocyanins. Red cabbage, radish, and black carrot were found to contain high amounts of acylated anthocyanins [119]. The cyanidin and delphinidin glycoside anthocyanins found in fruits of *Morus alba* are used as natural food coloring agents. These reddish-purple to purple pigments have strong antioxidant properties such as FRAP and DPPH [120,121]. *Morus alba* and its anthocyanins are highly suggested as a natural, functional food colorant due to their high antioxidant and color-enhancing characteristics since they can produce better color quality with value-added attributes for the product and are safer to take as compared to synthetics [122]. Himalayan flora offers a diverse range of anthocyanins, these anthocyanins can be extracted and stabilized for their uses as a natural coloring agent in food industries. Anthocyanins can be stabilized for application in food systems. Additions of co-pigment chemicals, such as polymers, phenolic compounds, and metals, are among the stabilizing methods. Various encapsulation processes, hard-panned candy-coating procedures for the colors blue, green, and brown, and the exclusion of O2 during production and storage can also be taken into consideration [123].

Anthocyanins have potential applications in the nutriment sector for the development of various fortified foods and beverages, which may have positive health effects [124]. Berries, grapes, red and purple vegetables, as well as their processed products, including drinks, are food sources rich in anthocyanins. (Wines, juices). Anthocyanin-rich feedstocks are used to obtain red wine which is an important source of antioxidants when consumed in moderate amounts. Anthocyanins hold great importance in wine research as they play a major role in affecting wine properties such as wine color, mouthfeel, aging, stability, and overall quality [125]. Himalayan sources of anthocyanins and their closely related species are already being used to prepare food and beverages with high nutritional value and functional characteristics. *Morus alba* is highly regarded as a delightful dessert fruit and is commonly utilized in making jelly, refreshing cold drinks, and alcoholic beverages. It is frequently incorporated into ice cream, gastriques, sorbet, and various baked goods, particularly pies. Mulberry leaves are commonly combined with other herbs such as ligustrum, chrysanthemum, and apricot seeds. They are used in various food and beverage applications such as wine, fruit juice, canned food, and jams. Mulberry leaves are especially useful in creating desserts, dairy products, shakes, and tea. They are also employed in the preparation of different types of beverages [126]. Shrubs belonging to the *Berberis genus* are part of the Berberidaceae family. They naturally grow in central and southern Europe, western Asia, and northwest Africa. The fruit called Zereshk is highly popular in Iran. It is commonly used in cooking and making jam, leading to a yearly production of about 22,000 tons of fresh, seedless barberries. These fruits are also used to make beverages, syrups, candy, and other sweet treats that are enjoyed in Iran. Additionally, the leaves and fruits are used to create food flavorings and herbal teas [127]. 

Numerous attempts have been made to fortify anthocyanins in real food systems. Bread fortified with 1%, 2%, and 4% of anthocyanin-rich black rice extract powder (ABREP) as a nutraceutical ingredient had digestion rates that were lowered by 12.8%, 14.1%, and 20.5%, respectively. These findings suggested that adding anthocyanins to bread could open new production possibilities for functional bread by reducing the rate of digestion and so improving consumer health [128]. It has been observed that using anthocyanins to fortify and/or color some foods has produced functional foods with health advantages. Adding extracts rich in anthocyanins to baked goods such as cookies, biscuits, and macarons can curb damage to the food during baking and increase its antioxidant capacity beyond that of synthetic additives, all without impacting the food’s acceptability [129,130,131,132]. Moreover, anthocyanins have proved to have great stability during storage in products such as kefir, yogurt, and various beverages, and might be thought of as ideal foods for anthocyanin fortification [133,134,135].

### 6.2. Health

Anthocyanins have significant effects on human health. Research has shown that the intake of these pigments reduces the rate of ailments of the heart and blood vessels, cancer, hyperlipidemia, and chronic diseases [136]. Anthocyanins have the capacity to terminate or neutralize free radicals and reactive chemical species, controlling signaling pathways, reducing the extent to pro-inflammatory factors, and lowering the occurrence of neurodegenerative diseases, cancer, and cardiovascular ailments [137]. Anthocyanins are known to provide a variety of health benefits, but researchers have paid more attention to the antioxidant properties of diets sufficient in anthocyanins and their role in improving cardiovascular health. *M. alba* root, also called San Baipi in traditional Chinese medicine, is commonly used to cure conditions such as cough, asthma, and similar ailments. In a study, fourteen compounds were extracted from *M. alba* and evaluated for their potential effects against HIV [138] Antioxidants may confer a beneficial impact on human health at several levels since free radical damage has a major function in the genesis of many chronic diseases. Traditionally, the leaves, fruits, and latex of *Ficus palmata* have been used to treat several disorders such as hypoglycemia, hyperlipidemia, gastrointestinal disorders, tumors, ulcers, diabetes, and fungal infections [139]. This plant has been documented in several scientific reports for its medicinal applications in treating a range of diseases and disorders. These include gastrointestinal issues, diabetes, tumors, hypoglycemia, ulcers, lipid level reduction, and antimicrobial properties [50]. In many in vitro models, anthocyanins have proven to be strong antioxidants and free radical scavengers. The anthocyanins that possess the highest antioxidant capacities among those tested are the 3 glucosides of delphinidin (gallocatechol structure) [140]. Existing clinical trials on this group of plants primarily focus on their effects on conditions related to cardiovascular diseases, associated risk factors, neurodegenerative diseases, and inflammation. One notable group of clinical trials conducted investigated a specific combination of *B. aristata* and Silybum marianum. This combination was chosen to address the low bioavailability of *B. aristata*, while *S. marianum* was included to enhance its absorption in the intestines. In a 52-week double-blind placebo-controlled study involving 136 obese patients with type-2 diabetes mellitus (T2DM) and metabolic syndrome, various parameters were analyzed. These parameters included fasting blood glucose, insulin levels, total cholesterol, HDL and LDL cholesterol, triglycerides, and body mass index [141,142,143]. The wild edible fruits of Fragaria nubicola, belonging to the Rosaceae family, are known for their significant content of antioxidants and polyphenolics. These fruits hold great potential as a valuable source for the nutraceutical and food industries.

Protection from oxidative stress is closely related to anthocyanin’s role in preventing cardiovascular diseases [144]. Anthocyanins have the capability to act upon different cells responsible for the occurrence of atherosclerosis which is one of the major causes of cardiovascular dysfunction [145]. It has been found that anthocyanins have anti-inflammatory as well as chemo preventive effects. Different cell cycles and growth-related pathways have been recognized as potential targets for anthocyanins used in in vitro and on lab animals. Red wine’s anthocyanin-rich fraction has been demonstrated to have a potency for inhibiting the growth of gastric adenocarcinoma (AGS) and HCT-15 human colon cancer cells [146,147]. Anthocyanins have already been found to assist in improving vision and weight management [148]. In a study conducted in 2000, oral doses of anthocyanins obtained from blackcurrant, depending on the dose, were competent in lowering the threshold for dark adaptation [149]. Bilberry anthocyanins when interacting with rhodopsin or phosphodiesterase improved night vision [150]. Anthocyanins also have a potency for regulating adipocyte function, thus, preventing obesity [151]. Anthocyanins also have antidiabetic and anti-obesity effects and act they act as neuroprotective agents. Numerous studies have demonstrated that anthocyanin consumption has a beneficial impact on human health [152]. 

### 6.3. Active and Intelligent Packaging Films

Although conventional had great importance in the historical development of food distribution networks, it is no more adequate due to the complexity of modern society. Innovative packaging with enhanced functionality is highly desired due to consumer demands for foods that have fewer chemical preservatives and are minimally processed, better regulation, food safety concerns, and globalized markets [153]. Conventional food packaging films are made of synthetic polymers, which are not biodegradable and can only shield food from certain external environmental factors such as moisture, light, air, microbes, and mechanical harm [154,155]. Therefore, researchers have given more attention to the development of active packaging technology that works by releasing functional components into the packaged food or the external environment to maintain its quality and extend shelf life. On the other side, intelligent packaging helps to monitor the freshness of the food during packaging. Functional components such as organic acids, enzymes, microbial toxins, natural extracts, and ionic compounds are deliberately included in the packaging material to develop these active packaging systems [156,157]. According to the legal definition of the EU (EC, 2009), intelligent packaging includes a component that permits the monitoring of the state of packed food or the environment around the food during transport and storage. Hence, intelligent packaging is a system that gives the user accurate and reliable details on the state of the food, the environment, and/or the integrity of the package. Intelligent packaging conveys information to the consumer based on its capacity to sense, detect, or record changes in the food product or its environment [158].

Researchers have recently become interested in the development of active and intelligent packaging by incorporating anthocyanins with biopolymers. These packaging films are developed by casting or extrusion methods. The matrix of anthocyanin-rich films can be several polysaccharides such as starch, cellulose, pectin, chitosan, and agar. In addition to that, proteins such as zein, soy protein, and soy protein isolate can be used [159]. The anthocyanin-added packaging film/label is found to be highly effective to determine the freshness of meat and fish products, dairy products, and fruits and vegetables.

The excellent antioxidant and antimicrobial properties of anthocyanins enable these films to keep food product quality high and prolong their shelf lives [160,161,162]. Free radicals and other reactive species play an important role in causing food spoilage and loss of nutrients [163]. Food packaging films having antioxidant activity can help extend the shelf life of food [164]. Thus, the addition of anthocyanin-rich extracts can significantly increase the films’ antioxidant activity [165]. Berberis plants have significant potential in the food processing industry, although there is limited research on their direct application in food products. One example is the use of seed oil and fruit extracts from *B. crataegina* to enhance the physical and functional properties of chitosan-based edible film. The resulting films were studied for their physical, chemical, and biological properties. The findings revealed that the chitosan-fruit extract film exhibited superior thermal stability, antimicrobial, antioxidant, and anti-quorum sensing activities compared to other films. These results suggest that incorporating *B. crataegina* fruit extract into a chitosan-based edible film can improve its overall characteristics, making it a valuable ingredient in food production [166]. Foodborne diseases have serious consequences and may result in mortality and economic loss due to spoilage. Transnational food supply can become a pathway for the spread of foodborne diseases. Therefore, the globalization of food supply is under threat of an outbreak of foodborne diseases [167]. To prevent pathogen growth and food spoiling, antimicrobial activity is a crucial characteristic of food packaging films prolonging the food’s shelf life [168]. Anthocyanins were found to have considerate antimicrobial potential [167]. The biopolymer-based film having no anthocyanin exhibited minimal to no antimicrobial activity. However, with the addition of anthocyanins-rich extracts, the antimicrobial nature of the films can be greatly increased [169]. A ternary blend film has been made using chitosan, gelatin, poly (vinyl alcohol), and *Duchesnea indica* extract (DIE) using a casting method. In compliance with the antimicrobial properties of anthocyanins and other phytochemicals, with the release of DIE from the film matrix, the film demonstrated a clear inhibitory action on *S. aureus*, up to 4.9 logs CFU/mL. Such films can be used as a coating material in fresh fruits and vegetables to extend their microbial safety [170]. Additionally, because anthocyanins can change their chemical structures and exhibit different colors depending on pH, anthocyanin-rich films can be used to evaluate the quality of packaged foods [171]. Further research is required on the preparation of anthocyanin-based active and intelligent packaging systems by utilizing Himalayan plants. 

### 6.4. Nutraceuticals and Functional Foods

Nutraceuticals are foods or components of foods that shows health benefits, such as the ability to treat or prevent disease. Because they avoid side effects and contain naturally occurring dietary supplements, nutraceuticals offer an advantage over pharmaceuticals [172]. Anthocyanins are gaining popularity as one of the most favorable ingredients in nutraceutical preparations. Many studies have been conducted on the nutraceutical properties of anthocyanins and anthocyanidin [173]. Anthocyanidins are aglycones from which anthocyanins are formed. These substances have a flavylium cation backbone that can be hydroxylated in a variety of ways to form various anthocyanidins (usually on carbons C3, C5, C6, and C7 as well as C3′, C4′, and C5′). The charged oxygen atom is found on the C ring of flavonoids, which preserves their ring nomenclature while having an oxonium group in their structure. Their brilliant colors such as red, blue, and purple, are caused by the buildup of these substances and their capacity to absorb light that is opposite to chlorophyll. Because it lessens the extremely appealing green coloring of some plants, this color variation may act as a defense strategy for those plants against possible herbivorous predators [174]. Due to the flavylium skeleton, which permits the delocalization of radical electrons on the sp2 orbitals of the oxonium group, anthocyanins have special properties. The oxidation of anthocyanins’ phenolic hydroxyl groups is intimately related to their antioxidant action. The stabilization of oxidation products resulting from one-electron oxidation and the production of semiquinones depend heavily on the para- and ortho- phenolic groups. The antioxidant qualities of anthocyanin extracts produced from various culinary and agricultural sources have been the subject of numerous investigations. To assess the radical scavenging properties of anthocyanins, these investigations frequently use techniques based on single electron transfer (SET) processes, hydrogen atom transfer (HAT), or a combination of the two. The DPPH and ABTS+ antioxidant tests are examples of SET and HAT assays, which involve direct electron transfer [175,176,177]. Anthocyanins have significant antioxidant, anti-inflammatory, anti-fibrotic, and anti-apoptotic properties and have been suggested for the prevention and treatment of several disorders [178]. Anthocyanin’s antidiabetic properties have been well investigated. Petunidin-3-O-p-coumaryl-rutinoside-5-O-glucoside is the major anthocyanin found in the extract of the Blue Congo variant of purple potato, in a study, it was found to reduce the fasting sugar levels in diabetic rats induced with streptozotocin [179]. In Zucker Diabetic Fatty rats, the mulberry fruit’s anthocyanin extract significantly lowered glucose levels, demonstrating its anti-diabetic effects [180]. Anthocyanin’s anticancer properties have been well-researched and examined [181]. The anti-cancer activities of Vitelotte potato (*Solanum tuberosum* L.) anthocyanins have been well investigated in the research. The implications for food consumption and as functional food ingredients for potential cancer prevention and treatment make these findings interesting [182]. Studies have also shown that anthocyanins in purple tea had anti-inflammatory, anticancer, and antioxidant properties [183]. The Himalayan region’s wild edible fruit plants are a rich source of diverse antioxidant bioactive compounds including anthocyanins. The food and flavor industries are looking for novel food ingredients to create dietary supplements. Therefore, these plants have the potential to serve as sources of anthocyanins for nutraceutical development [40]. *Berberis lycium* berries are a rich source of anthocyanins. They are consumed in raw form, particularly by the rural population [184]. In a study, the phytochemical, antioxidant, and antimicrobial profiling of *Berberis asiatica* fruit was conducted in four different processed forms (dried fruit, pulp, juice, and pomace). It was concluded that fruit can be utilized for nutraceutical development, and pomace works best for fruit waste utilization in food and nutraceutical applications [185]. In another study, the fruits of *Ficus palmata* were investigated using different extraction methods for their comparative phytochemical and biological activities. The results indicated that the fruit is a reservoir of polyphenols such as anthocyanins and may be suggested for the development of new nutraceuticals and related products [186]. These studies indicate that the underutilized anthocyanin-rich Himalayan plants are potential candidates for emerging nutraceutical industries. The sustainable utilization of these plants can help in the nutritional security of consumers and would also benefit the local people.

## 7. Conclusions and Future Perspectives

Anthocyanins are natural colorants that are gaining popularity due to their diverse color palette as well as safe and favorable health effects. Anthocyanins have a high potential for application in food, pharmaceutical, cosmetic, and related industries. Anthocyanin can be isolated and purified from an endless number of natural resources, but the Himalayan sources of anthocyanin are relatively undiscovered. Most anthocyanin research is currently focused on identifying different sources of anthocyanin, as well as purification and extraction. Natural food colorants are recently being preferred by consumers since they have fewer adverse effects than synthetic/artificial substances. Himalayan plants are rich sources of anthocyanins. Anthocyanins have wide applications in the health, nutraceuticals, food, and other sectors. Anthocyanin has been emerging as an excellent choice for making intelligent packaging film as a freshness indicator. Anthocyanin-based food colorants are universally authorized, even though there are significant regulatory variances between countries. Human-nature relationships have long been woven with the importance of anthocyanins in healing a variety of ailments. However, a better knowledge of the processes and mode of action that led to these effects has yet to be developed. A collaborative effort is currently required by academia, industry, and consumer welfare organizations to examine the benefits of these pigments and develop sustainable food colorants for human health and well-being. Moreover, the stability of anthocyanin and its degradation is a challenge that needs to be improved. Ultimately, the potential and identification of these Himalayan sources of anthocyanins, in addition to accurate study and utilization may help achieve sustainable development.

## Figures and Tables

**Table 1:** Classification of different Himalayan plant sources of anthocyanins (H = Hindi, E = English, NA = not available).

Name of the Species	Family	Common Name	References
*Berberis asiatica*	Berberidaceae	H: Chitra/Chotra E: Indian Barberry, Tree Turmeric	[26]
*Morus alba*	Moraceae	H: Shahtoot E: Mul berry/Silkworm Mulberry	[27]
*Berberis lycium*	Berberidaceae	E: Indian Barberry, H: Kashmal	[28]
*Myrica esculenta*	Myricaceae	E: Box berry H: Kaphal	[29]
*Duchesnea indica*	Rosaceae	H: Kiphaliya E: Indian strawberry/Mock strawberry	[26]
*Lycium ruthencium*	Solanaceae	E: Black wolfberry	[30]
*Fragaria nubicola*	Rosaceae	E: Himalayan strawberry	[31]
*Ficus palmata*	Moraceae	H: Abjiri/Bedu E: Wild Fig	[26]
*Gaultheria trichophylla*	Ericaeae	E: Himalayan snowberry	[32]
*Genus begonia*	Begoniacece	NA	[33]

## Data Availability

Data is contained within the article.
